# Giving Drugs a Second Chance: Antibacterial and Antibiofilm Effects of Ciclopirox and Ribavirin against Cystic Fibrosis *Pseudomonas aeruginosa* Strains

**DOI:** 10.3390/ijms23095029

**Published:** 2022-04-30

**Authors:** Giovanni Di Bonaventura, Veronica Lupetti, Simone De Fabritiis, Alessandra Piccirilli, Annamaria Porreca, Marta Di Nicola, Arianna Pompilio

**Affiliations:** 1 Department of Medical, Oral and Biotechnological Sciences, “G. d’Annunzio” University of Chieti-Pescara, 66100 Chieti, Italy; veronica.lupetti@unich.it (V.L.); annamaria.porreca@unich.it (A.P.); marta.dinicola@unich.it (M.D.N.); arianna.pompilio@unich.it (A.P.); 2 Center of Advanced Studies and Technology (CAST), “G. d’Annunzio” University of Chieti-Pescara, 66100 Chieti, Italy; simone.defabritiis@unich.it; 3 Department of Medicine and Aging Sciences, “G. d’Annunzio” University of Chieti-Pescara, 66100 Chieti, Italy; 4 Department of Biotechnological and Applied Clinical Sciences, University of L’Aquila, 67100 L’Aquila, Italy; alessandra.piccirilli@univaq.it

**Keywords:** drug repurposing, cystic fibrosis, *Pseudomonas aeruginosa*, biofilm

## Abstract

Drug repurposing is an attractive strategy for developing new antibacterial molecules. Herein, we evaluated the in vitro antibacterial, antibiofilm, and antivirulence activities of eight FDA-approved “non-antibiotic” drugs, comparatively to tobramycin, against selected *Pseudomonas aeruginosa* strains from cystic fibrosis patients. MIC and MBC values were measured by broth microdilution method. Time–kill kinetics was studied by the macro dilution method, and synergy studies were performed by checkerboard microdilution assay. The activity against preformed biofilms was measured by crystal violet and viable cell count assays. The effects on gene expression were studied by real-time quantitative PCR, while the cytotoxic potential was evaluated against IB3-1 bronchial CF cells. Ciclopirox, 5-fluorouracil, and actinomycin D showed the best activity against *P. aeruginosa* planktonic cells and therefore underwent further evaluation. Time–kill assays indicated actinomycin D and ciclopirox, contrarily to 5-fluorouracil and tobramycin, have the potential for bacterial eradication, although with strain-dependent efficacy. Ciclopirox was the most effective against the viability of the preformed biofilm. A similar activity was observed for other drugs, although they stimulate extracellular polymeric substance production. Ribavirin showed a specific antibiofilm effect, not dependent on bacterial killing. Exposure to drugs and tobramycin generally caused hyperexpression of the virulence traits tested, except for actinomycin D, which downregulated the expression of alkaline protease and alginate polymerization. Ciclopirox and actinomycin D revealed high cytotoxic potential. Ciclopirox and ribavirin might provide chemical scaffolds for anti-*P. aeruginosa* drugs. Further studies are warranted to decrease ciclopirox cytotoxicity and evaluate the in vivo protective effects.

## 1. Introduction

In cystic fibrosis (CF) patients, the mutation of the CF transmembrane conductance regulator gene leads to an accumulation of dry and sticky airway secretions, creating the perfect environment for the onset of bacterial pulmonary infections [1]. The altered microenvironment of the CF lung counteracts the inflammation in clearing the infection, thus causing the progression of pulmonary disease towards bronchiectasis and finally death [2].

*Pseudomonas aeruginosa* is the predominant pathogen, especially in adulthood [3]. Although the aerosolization of tobramycin into CF patients’ airways improves outcomes, the lungs of CF patients, even those receiving antibiotic therapy, are persistently colonized by *P. aeruginosa* [3,4]. Indeed, this microorganism cannot be eradicated because of its ability to grow as biofilm in the CF airway, a functional consortium of sessile bacteria enclosed in an extracellular matrix, making them significantly more tolerant to antimicrobials than with planktonic counterparts [5,6,7]. In addition, another *P. aeruginosa* adaptative reply to the airway of CF patients is the conversion to a mucoid phenotype due to an overproduction of alginate, leading to the generation of a thicker extracellular polysaccharide matrix [3,8].

As a result, the decreasing number of effective antibiotics raises the urgent need to develop new molecules that possibly target cells within the biofilm and avoid selecting resistant strains. However, the traditional drug discovery process is costly and lengthy, requiring years of experimentation followed by extensive clinical trials [9]. 

The “drug repurposing” approach recently proposed to reduce the drug discovery time frame is an attractive alternative. This strategy is based on using known and approved drugs for a medical indication other than the one for which it was developed [10]. It can also include using drugs that have reached phase II or III of clinical trials that demonstrate no efficacy for a particular indication but have shown good safety [10]. Since their toxicity and pharmacokinetics have already been studied, the “repurposed” drugs can bypass some clinical trials, save time, and reduce costs [11]. This strategy is a promising tool in treating bacterial infections, as many molecules have secondary mechanisms of action that allow them to be effective against many pathogens. Several non-antimicrobial drugs have demonstrated antibiotic activity [12], but none are currently used in antibacterial therapy.

In this frame, the main aim of the present study was to assess the in vitro activity of eight FDA-approved “non-antibiotic” drugs—namely ribavirin (antiviral), toremifene (nonsteroidal antiestrogen), oxyclozanide (anthelmintic), meloxicam (nonsteroidal anti-inflammatory), 5-fluorouracil (antineoplastic), actinomycin D (antineoplastic), furosemide (diuretic), and ciclopirox (antifungal)—against a set of selected *P. aeruginosa* strains isolated from CF patients. These in-use pharmacological agents, encompassing a wide variety of different chemical structures and mechanisms of action, were selected because they were previously found to have some direct antibacterial activity (Table 1). The antibacterial, antibiofilm, and antivirulence potential of the non-antibiotic drugs were evaluated compared to tobramycin.

## 2. Results

### 2.1. Selection of P. aeruginosa Strains

**Figure 1:**
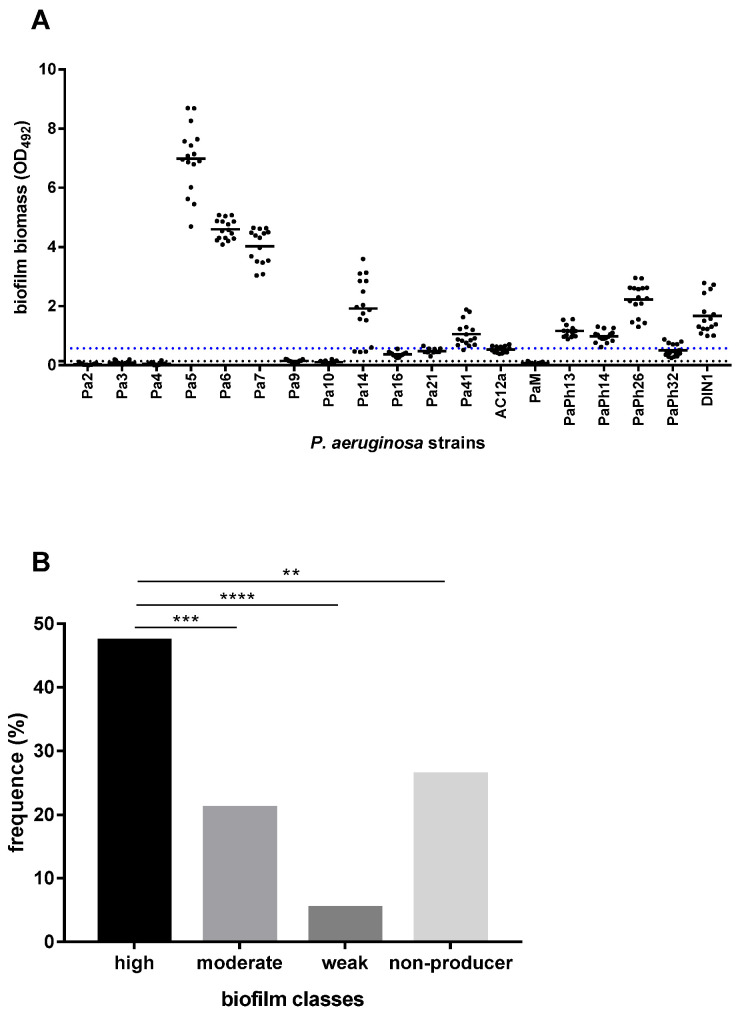
Biofilm formation by 19 *P. aeruginosa* strains from CF patients. The amount of biofilm formed on polystyrene following 24 h of incubation at 37 °C was measured by the microtiter plate crystal violet method. Each strain was tested, in quadruplicate, on four different occasions (*n* = 16). (**A**) Results were subtracted by negative control (OD_492_ = 0.092) and shown as a scatter plot, with the horizontal solid line indicating the mean OD value. The horizontal dotted black line shows the cut-off value for biofilm formation (ODc = mean + 3 x standard deviation of negative control wells; ODc = 0.143), whereas the blue one indicates the cut-off value for high biofilm-former class (OD_492_ > 0.572). (**B**) According to Stepanović et al. [21], each strain was assigned to one of the following groups: non-biofilm-former (OD_492_ < 0.143), weak biofilm-former (0.143 < OD_492_ ≤ 0.286), moderate biofilm-former (0.286 < OD_492_ ≤ 0.572), and high biofilm-former (OD_492_> 0.572). Results are shown as the percentage of distribution of each group. Statistical significance at Fisher’s exact test: ** *p* < 0.01, *** *p* < 0.001, **** *p* < 0.0001.

First, a collection of 19 *P. aeruginosa* strains from CF patients was screened for the ability to form biofilm on polystyrene using a microtiter plate crystal violet assay, and the results are shown in Figure 1. 

Most strains (14 out of 19, 73.7%) were able to form a biofilm, although with significant differences for biofilm biomass, measured as optical density at 492 wavelength (OD_492_) (OD_492_ range: 0.029–6.986; *p* < 0.0001, ordinary one-way ANOVA test) (Figure 1A). The highest biofilm biomass was produced by *P. aeruginosa* Pa5 (OD_492_, mean ± SD: 6.986 ± 1.124; *p* < 0.0001 vs. other strains), Pa6 (4.593 ± 0.340; *p* < 0.0001 vs. other strains but Pa7), and Pa7 (4.021 ± 0.581; *p* < 0.0001 vs. other strains) strains (Figure 1A). Considering the efficiency in forming biofilm measured according to the criteria proposed by Stepanović et al. [21], the prevalence of high biofilm-former (HBF) strains was significantly higher than in other groups (9 out of 19, 47.4%; Pa5, Pa6, Pa7, Pa14, Pa41, PaPh13, PaPh14, PaPh26, and DIN1 strains; *p* < 0.01, vs. other groups), followed by moderate biofilm-producer (MBF) strains (4 out of 19, 21%; Pa16, Pa21, AC12a, and PaPh32 strains), and weak biofilm-former (WBF) strains (1 out of 9, 11.1%; Pa9 strain) (*p* < 0.01, vs. other groups) (Figure 1B). Five strains (Pa2, Pa3, Pa4, Pa10, and PaM) could not form biofilm.

Next, the susceptibility of 19 *P. aeruginosa* strains to eight anti-pseudomonal antibiotics was measured using a disk diffusion agar test, and the results are summarized in Table 2. Amikacin and tobramycin were the most active antibiotics showing a susceptibility rate of 63.1%, followed by colistin (52.6%), netilmicin (31.6%), and ceftazidime (5.2%). Contrarily, ticarcillin and piperacillin/tazobactam showed no activity against the strains tested. According to Magiorakos et al. [22], all *P. aeruginosa* strains but two (Pa14 and Pa16) were classified as multi-drug resistant (MDR).

Based on the findings from biofilm formation and antibiotic susceptibility assays, we selected six *P. aeruginosa* strains (Pa5, Pa6, Pa7, Pa41, PaPh32, and DIN1) because they are representative of both HBF and MDR phenotypes. Furthermore, Pa41 and PaPh32 strains showed a mucoid phenotype due to the overproduction of the polyanionic exopolysaccharide alginate that is often associated with a poor prognosis for the CF patient [23].

### 2.2. Antibacterial Activity of FDA-Approved Drugs against P. aeruginosa Planktonic Cells

MIC and MBC values of the FDA-approved drugs with medical indication other than “antibiotic” were measured against the selected *P. aeruginosa* strains, comparatively to tobramycin, by the broth microdilution technique, and the results are shown in Table 3.

As expected, tobramycin showed the highest activity (MIC: 0.5–64 µg/mL; MBC: 0.5–>64 µg/mL), showing a bactericidal effect since the killing quotient (i.e., MBC/MIC ratio) was always ≤ 4. Among the “non-antibiotic” drugs, ciclopirox (MIC: 128–512 µg/mL; MBC > 1024 µg/mL), 5-fluorouracil (MIC: 128–>1024 µg/mL; MBC: 1024–>1024 µg/mL), and actinomycin D (MIC, MBC: 133–>266 µg/mL) were the most active with a strain-dependent efficacy. Conversely, ribavirin, oxyclozanide, meloxicam and furosemide (MIC, MBC > 1024 µg/mL), and toremifene (MIC, MBC > 330 µg/mL) showed no activity at the concentrations tested. Based on these results, ciclopirox, 5-fluorouracil, and actinomycin underwent further antibacterial characterization, along with the comparator tobramycin.

### 2.3. Killing Kinetics

**Figure 2:**
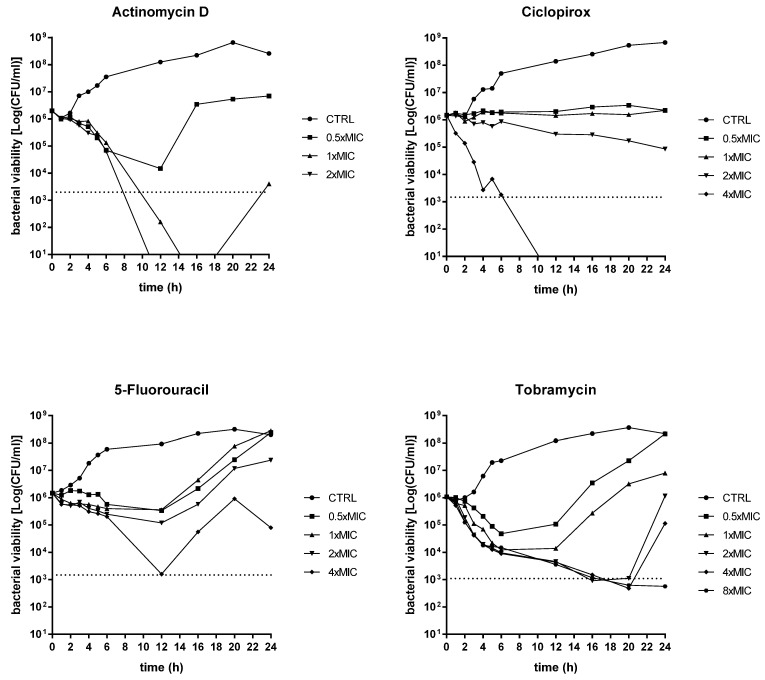
Time–kill kinetics against *P. aeruginosa* PaPh32. The kinetics of repurposed drugs was assessed, comparatively to tobramycin, over 24 h in a liquid medium. *P. aeruginosa* PaPh32 was chosen as representative of tobramycin-resistant strains. Each drug was tested at MIC value (actinomycin D: 133 mg/L; ciclopirox: 256 mg/L; 5-fluorouracil: 512 mg/L; tobramycin: 64 mg/L), its fractions and multiples, compatibly with the drugs’ solubility. The dotted line indicates bactericidal activity, defined as a ≥ 3 Log (CFU/mL) reduction of the initial inoculum size. The limit of detection was 10 CFU/mL.

**Figure 3:**
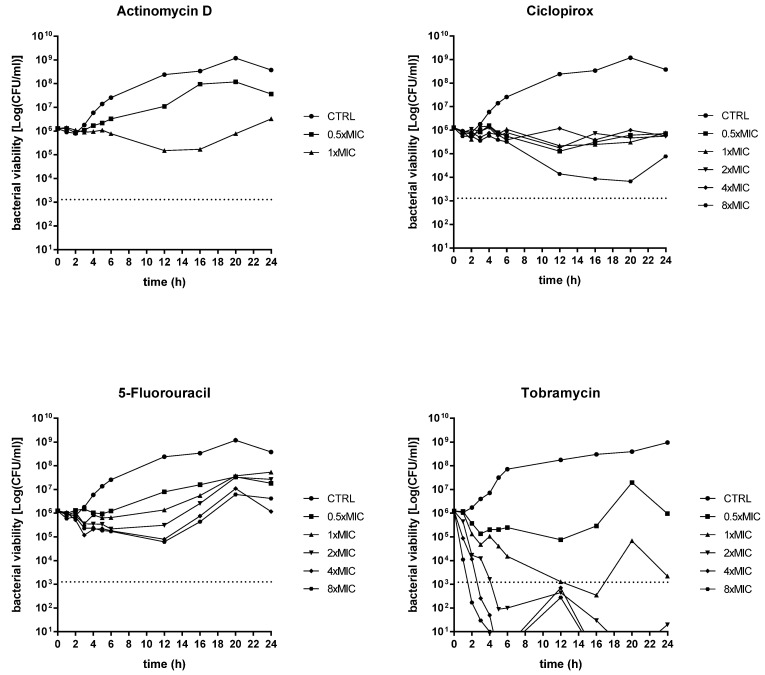
Time–kill kinetics against *P. aeruginosa* Pa7. The kinetics of repurposed drugs was assessed, comparatively to tobramycin, over 24 h in a liquid medium. *P. aeruginosa* Pa7 was chosen as representative of tobramycin-susceptible strains. Each drug was tested at MIC value (actinomycin D: 266 mg/L; ciclopirox: 128 mg/L; 5-fluorouracil: 128 mg/L; tobramycin: 2 mg/L), its fractions and multiples, compatibly with the drugs’ solubility. The dotted line indicates bactericidal activity, defined as a ≥ 3 Log (CFU/mL) reduction of the initial inoculum size. The limit of detection was 10 CFU/mL.

The bactericidal or bacteriostatic properties of ciclopirox, 5-fluorouracil, and actinomycin were investigated, compared to tobramycin, by killing kinetics. We choose *P. aeruginosa* PaPh32 (Figure 2) and Pa7 (Figure 3) as representative of tobramycin-resistant and -susceptible strains, respectively.

An evident dose-dependent activity was observed only in the case of actinomycin D, although with different effects depending on the strain tested. It always had a bacteriostatic result towards the Pa7 strain, whereas it yielded a bactericidal effect—i.e., greater than 3 Log-fold decrease in CFUs, equivalent to 99.9% killing of the inoculum, was observed—towards the PaPh32 strain when tested at 1x (after 11 h of exposure) and 2xMIC (after 8 h of exposure). Actinomycin D caused a viability decrease of PaPh32 below the limit of detection (LOD) when tested at 1x (after 14 h of incubation) and 2xMIC (after 11 h of incubation), while a regrowth was found at 1xMIC. A similar trend was observed for ciclopirox that exerted bactericidal activity only when tested towards the PaPh32 strain after 7 h exposure at 4xMIC. The worst activity was shown by 5-fluorouracil, yielding a bacteriostatic effect over 24 h, regardless of the strain tested. By comparison, tobramycin had a bactericidal result against both strains, although to different extents. When tested towards PaPh32, it was bactericidal only at 4x and 8xMIC, although a rapid regrowth was observed in both cases. Contrarily, the effect was more rapid against Pa7, causing a 3 Log reduction within 5 h of incubation at 4x and 8xMIC and after 16 h of incubation at 1xMIC. The cell viability decreased below LOD at concentrations of 2x, 4x, and 8xMIC, although 2xMIC allowed Pa7 regrowth. Overall, the findings from time–kill assays showed that actinomycin D and ciclopirox have the potential for bacterial eradication, although their activity is strain-dependent. Contrarily, 5-fluorouracil showed a bacteriostatic effect regardless of the strain tested.

### 2.4. Synergy Tests

The activity of tobramycin in combination with actinomycin D, ciclopirox, and 5-fluorouracil was evaluated using a checkerboard assay. *P. aeruginosa* Pa7 and PaPh32 strains were selected because they are representative of tobramycin-susceptible and -resistant phenotypes, respectively (Table 4).

FICi values indicated additivity, regardless of strain and combination, with the best value of 0.56 for tobramycin + ciclopirox combination against Pa7 strain.

### 2.5. In Vitro Activity against Preformed Biofilms

**Figure 4:**
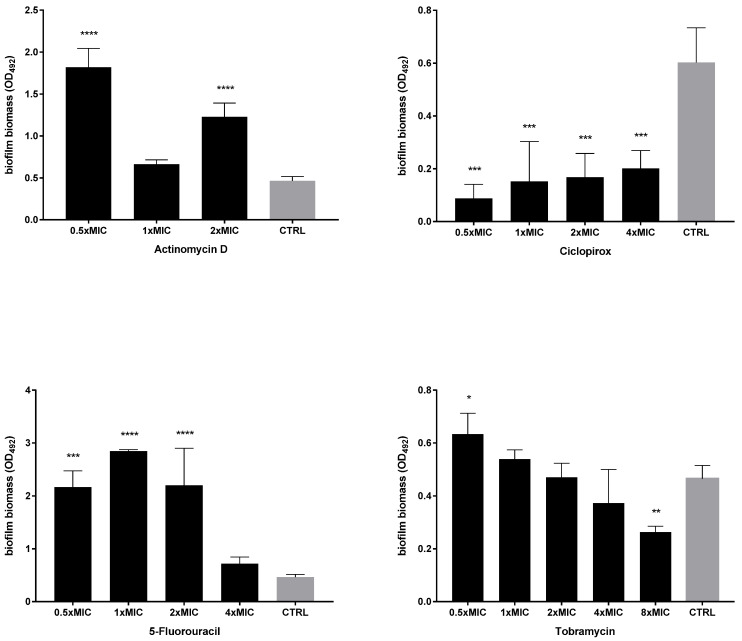
Dispersal activity against preformed biofilms by *P. aeruginosa* PaPh32. The efficacy of actinomycin D, ciclopirox, and 5-fluorouracil to disperse 24 h mature biofilms by *P. aeruginosa* PaPh32 was assessed, comparatively to tobramycin, using crystal violet assay. *P. aeruginosa* PaPh32 was chosen as representative of tobramycin-resistant strains. Each drug was tested at 0.5x and multiples of MIC value. Results were expressed as mean + SD of the residual biofilm biomass (OD_492_) after 24 h of exposure. Control samples (CTRL) were not exposed to the drug. Statistical significance at ordinary one-way ANOVA + Holm–Sidak’s multiple comparisons post-test: * *p* < 0.05, ** *p* < 0.01, *** *p* < 0.001, **** *p* < 0.0001 vs. CTRL.

**Figure 5:**
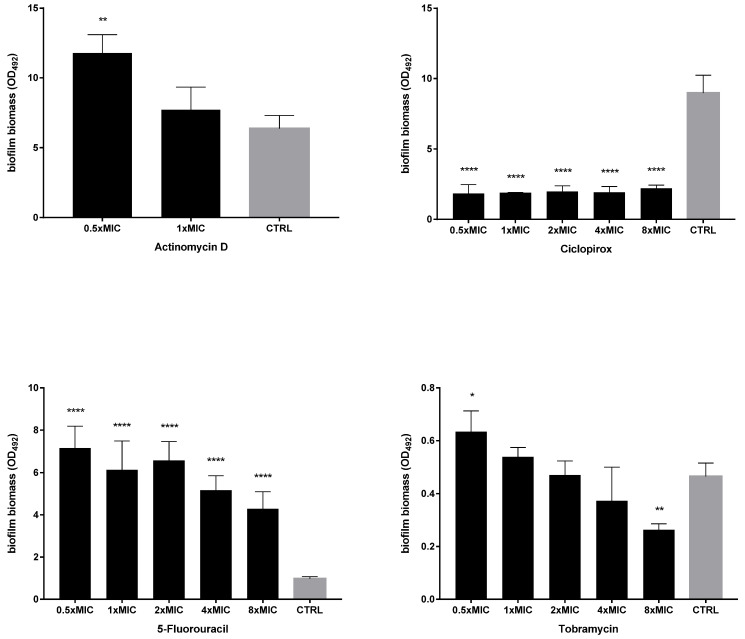
Dispersal activity against preformed biofilms by *P. aeruginosa* Pa7. The efficacy of actinomycin D, ciclopirox, and 5-fluorouracil to disperse 24 h mature *P. aeruginosa* Pa7 biofilms was assessed, comparatively to tobramycin, using crystal violet assay. *P. aeruginosa* Pa7 was chosen as representative of tobramycin-susceptible strains. Each drug was tested at 0.5x and multiples of MIC value. Results were expressed as mean + SD of the residual biofilm biomass (OD_492_) after 24 h of exposure. Control samples (CTRL) were not exposed to the drug. Statistical significance at ordinary one-way ANOVA + Holm–Sidak’s multiple comparisons post-test: * *p* < 0.05, ** *p* < 0.01, **** *p* < 0.0001 vs. CTRL.

Ciclopirox, 5-fluorouracil, and actinomycin D were tested, comparatively to tobramycin, for dispersal and killing activities against 24 h mature biofilms by *P. aeruginosa* PaPh32 (Figure 4) and Pa7 (Figure 5).

The activity of each drug against mature biofilm was not dependent on the strain tested. Ciclopirox was the most active, causing a significant reduction of biofilm biomass, regardless of concentration and strain tested. Conversely, the exposure to 5-fluorouracil and actinomycin D never caused biofilm reduction but even stimulated its biomass, although at different extents depending on strain and concentration, particularly in the case of 5-fluorouracil. Tobramycin significantly reduced biofilm biomass formed by both strains only when tested at the maximum concentration of 8xMIC, whereas at 0.5xMIC it constantly stimulated biomass formation.

**Figure 6:**
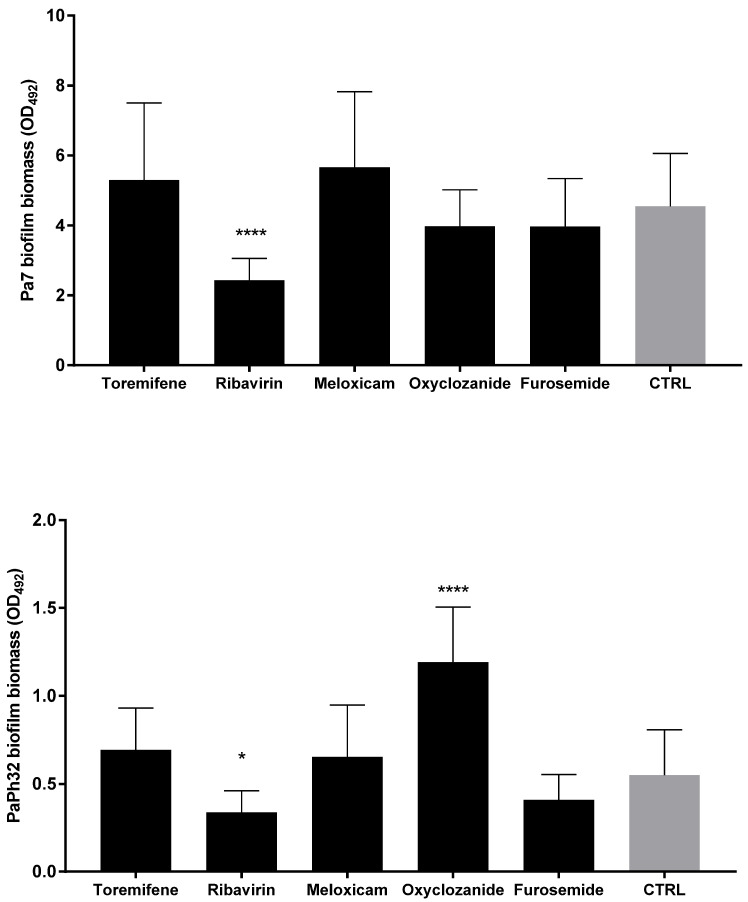
Biofilm dispersal activity of drugs not active against planktonic cells. The efficacy of drugs in disrupting 24 h mature biofilms was assessed using a crystal violet assay. Each drug was tested at the maximum concentration tested in MIC assays: 1.024 µg/mL for all drugs except for Toremifene (256 µg/mL). Results are expressed as mean + SD of the residual biofilm biomass (OD_492_) after 24 h of exposure. Control samples (CTRL) were not exposed to the drug. Statistical significance at ordinary one-way ANOVA + Holm–Sidak’s multiple comparisons post-test: * *p* < 0.05, **** *p* < 0.0001 vs. CTRL.

Since the antibiofilm effect could be specific and not related to antibacterial activity, the dispersal activity was also evaluated for drugs, which were not active against planktonic *P. aeruginosa* cells (Figure 6). Ribavirin was the only drug able to significantly decrease biofilm biomass, effective against both strains tested, although to different extents (biomass removal: 53.5% and 35.1%, respectively, for Pa7 and PaPh32; *p* < 0.001). Conversely, exposure to oxyclozanide significantly improved biofilm formation by about 100%, although only in the case of the PaPh32 strain.

**Figure 7:**
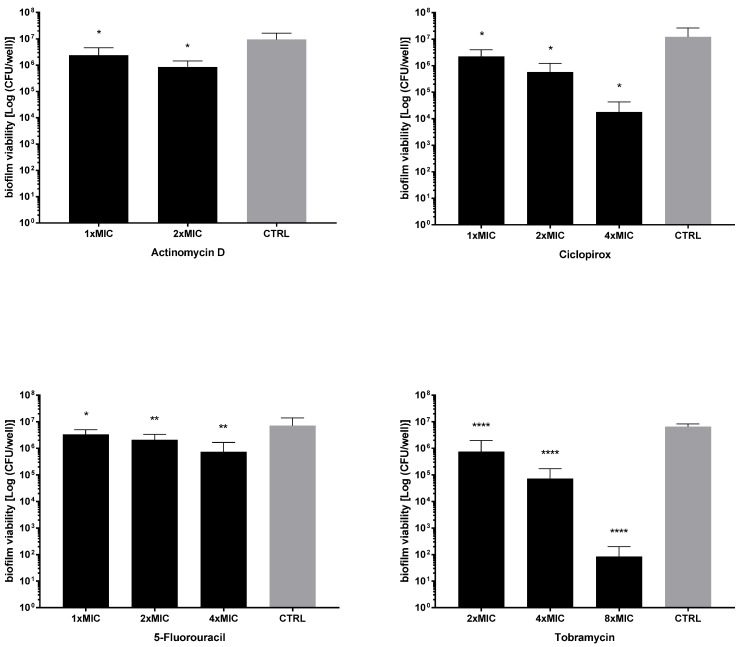
Killing activity against preformed biofilms by *P. aeruginosa* PaPh32. The efficacy of actinomycin D, ciclopirox, and 5-fluorouracil on the viability of 24 h mature biofilms by *P. aeruginosa* PaPh32 was assessed, comparatively to tobramycin, using cell viable count assay. Each drug was tested at multiples of MIC value. Results are expressed as mean + SD of the residual biofilm viability [Log (CFU/well)] after 24 h of exposure. Control samples (CTRL) were not exposed to the drug. Statistical significance at ordinary one-way ANOVA + Holm–Sidak’s multiple comparisons post-test: * *p* < 0.05, ** *p* < 0.01, **** *p* < 0.0001 vs. CTRL.

**Figure 8:**
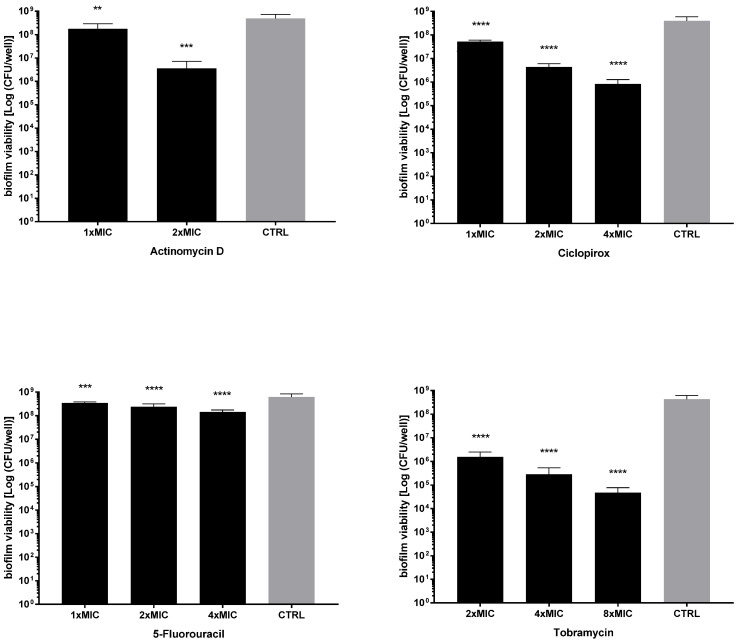
Killing activity against preformed biofilms by *P. aeruginosa* Pa7. The efficacy of actinomycin D, ciclopirox, and 5-fluorouracil on the viability of 24 h mature biofilms by *P. aeruginosa* Pa7 was assessed, comparatively to tobramycin, using cell viable count assay. Each drug was tested at multiples of MIC value. Results are expressed as mean + SD of the residual biofilm viability [Log (CFU/well)] after 24 h of exposure. Control samples (CTRL) were not exposed to the drug. Statistical significance at ordinary one-way ANOVA + Holm–Sidak’s multiple comparisons post-test: ** *p* < 0.01, *** *p* < 0.001, **** *p* < 0.0001 vs. CTRL.

Next, the activity of each drug against the viability of preformed biofilms was tested after exposure to MIC and its multiples, and the results are summarized in Figure 7 and Figure 8. All drugs caused a significant reduction of biofilm viability, regardless of strain and concentration tested, although at different levels. Ciclopirox was the most active, showing a dose-dependent activity regardless of the strain considered (viability reduction; Pa7: 98.7% at 2xMIC, and 99.8% at 4xMIC; PaPh32: 97.5% at 2xMIC, and 99.9% at 4xMIC). Tobramycin exhibited comparable activity to ciclopirox, causing a significant, concentration-dependent reduction ranging from 99.7% (2xMIC) to >99.9% (8xMIC) for Pa7 and from 88.5% (2xMIC) to >99.99% (8xMIC) for PaPh32.

Our findings indicated ciclopirox as the most effective against mature biofilms, active on biofilm biomass—consisting of extracellular polymeric substance (EPS) and cells—and viability. Although active on biofilm viability, the other drugs probably stimulate EPS production, as suggested by increased biofilm biomass after exposure. Of interest is the specific activity exhibited by ribavirin, which is not dependent on bacterial killing.

### 2.6. Effect on P. aeruginosa Virulence Genes Expression

**Figure 9:**
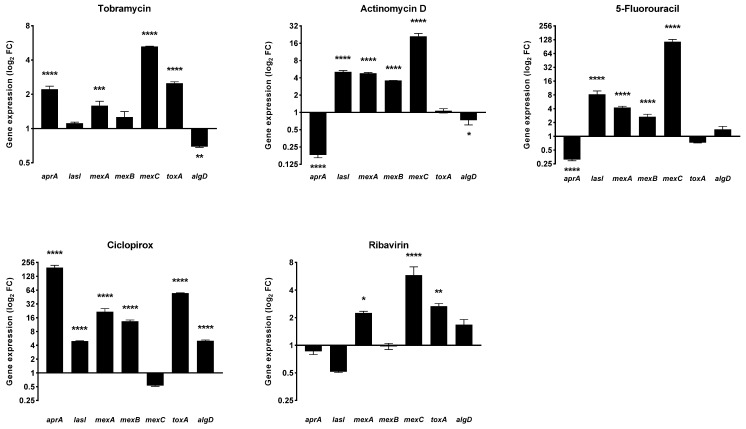
Effect of drugs exposure on the expression of selected *P. aeruginosa* virulence genes. The effects of exposure to actinomycin D, 5-fluorouracil, ciclopirox, and ribavirin on the expression levels of *P. aeruginosa* PaPh32 virulence genes *aprA* (alkaline protease), *lasI* (quorum sensing), *mexA*, *mexB,* and *mexC* (efflux pumps), *toxA* (exotoxin A), and *algD* (alginate) were assessed, comparatively to tobramycin, by real-time RT-qPCR assay. Each drug was tested at 1/4xMIC for 20 h, while controls (CTRL) were not exposed to the drug. The relative expression of each gene was normalized on the housekeeping *proC* gene. Results are shown as means + SDs (*n* = 6) of fold change (FC: 2^−^^ΔΔ^^ct^) on a log_2_ scale. Statistical significance at ANOVA followed by Tukey’s multiple comparisons post-test: * *p* < 0.05, ** *p* < 0.01, *** *p* < 0.001, and **** *p* < 0.0001 vs. CTRL.

The effect of 20 h of exposure to actinomycin D, 5-fluorouracil, ciclopirox, and ribavirin at 1/4xMIC on the expression of selected virulence genes of *P. aeruginosa* PaPh32 was evaluated, comparatively to tobramycin, by real-time RT-qPCR, and the results are shown in Figure 9.

The gene expression pattern was dependent on the drug and gene considered. The pattern observed after exposure to actinomycin D and 5-fluorouracil was nearly the same. Both drugs downregulated *aprA*, codifying for the alkaline protease (fold change: −5.53 for both; *p* < 0.001 vs. unexposed control), whereas they upregulated the expression of the QS mediator *lasI* (fold change: 5.07 and 8.19, respectively; *p* < 0.001) and all efflux pump-related genes *mexA* (fold change: 4.77 and 4.22, respectively; *p* < 0.001), *mexB* (fold change: 3.57 and 2.66, respectively; *p* < 0.001), and *mexC* (fold change: 20.95 and 115.04, respectively; *p* < 0.001). In addition, actinomycin D decreased the expression of *algD* (fold change: −1.38; *p* < 0.05), codifying for GDP-mannose 6-dehydrogenase and involved in the alginate polymerization. The exposure to ciclopirox caused the worst expression pattern, namely the hyperexpression of all genes but *mexC*. Finally, ribavirin significantly increased *mexA*, *mexC*, and *toxA* genes.

It is worth noting that the *aprA* and *toxA*—respectively codifying for protease and exotoxin A, the main *P. aeruginosa* virulence factors—were both upregulated (fold change: 2.12 and 1.44, respectively; *p* < 0.001 and *p* < 0.01, respectively) in the case of tobramycin. Exposure to tobramycin also induced upregulation of *mexA* (fold change: 1.58; *p* < 0.001) and *mexC* (fold change: 5.25; *p* < 0.001), whereas *algD* was down-expressed (fold change: −1.44; *p* < 0.01).

**Figure 10:**
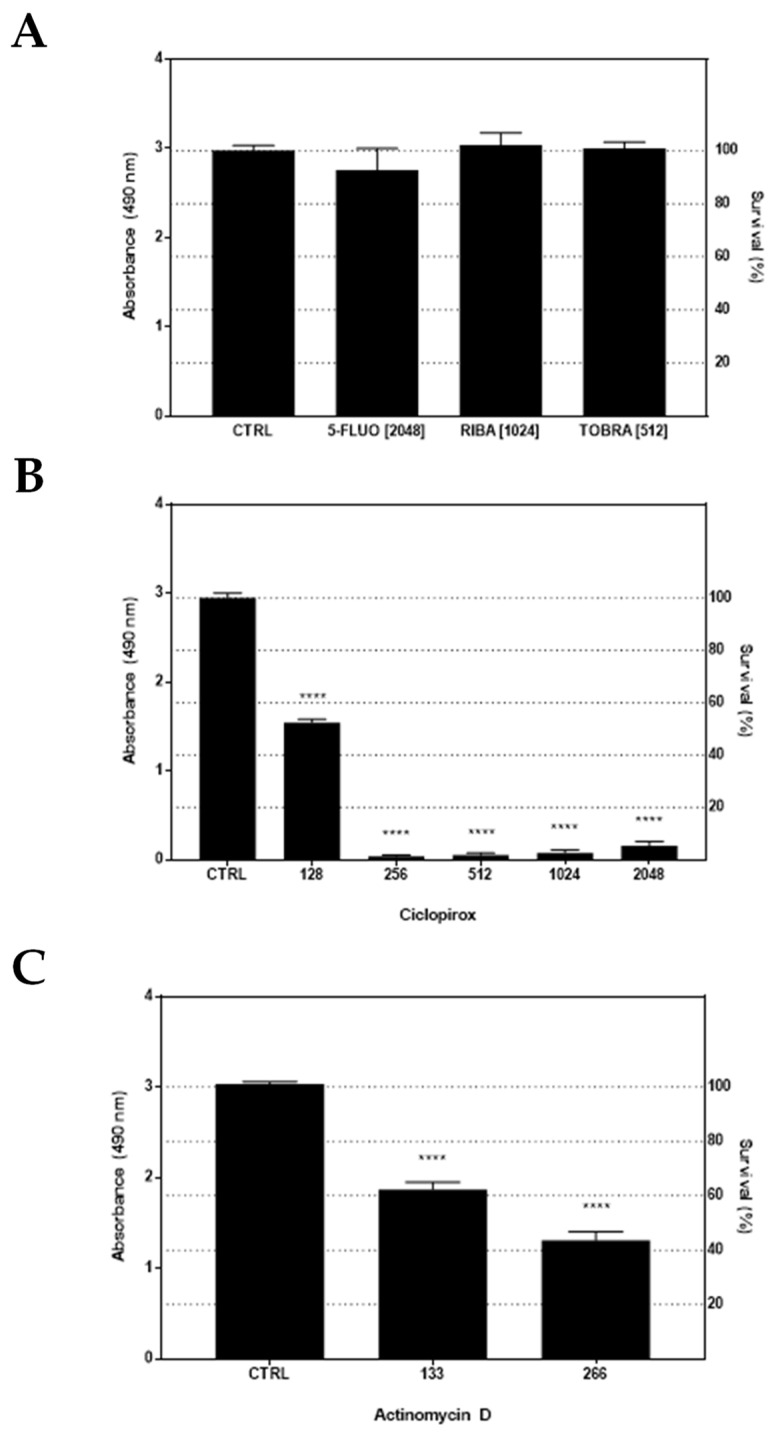
In vitro cytotoxicity against IB3-1 cells. (**A**) IB3-1 cell monolayers were initially exposed for 24 h to each drug at the highest biologically active concentration: 5-fluorouracil (5-FLUO, 2048 µg/mL), ribavirin (RIBA, 1024 µg/mL), tobramycin (TOBRA, 512 µg/mL), ciclopirox (2048 µg/mL), and actinomycin D (266 µg/mL). (**B**,**C**) Being toxic at the highest concentration, ciclopirox and actinomycin D were also evaluated at lower concentrations. The cell viability was measured by an MTS tetrazolium-based colorimetric assay and expressed as mean + SD absorbance at 490 nm (left *Y*-axis) and percentage of survival (right *Y*-axis) vs. CTRL (untreated cells). Statistical significance at one-way ANOVA + Holm–Sidak’s multiple comparisons post-test: **** *p* < 0.0001 vs. CTRL.

The cytotoxic potential of actinomycin D, 5-fluorouracil, ciclopirox, and ribavirin was investigated, in comparison with tobramycin, using a cell-based MTS assay. Each drug was tested at the maximum concentration active against planktonic and/or biofilm *P. aeruginosa* cells (Figure 10).

MTS tetrazolium-based colorimetric assay showed 5-fluorouracil, ribavirin, and tobramycin were not toxic for IB3-1 cells, allowing a cell growth comparable to untreated control cells (Figure 10A). On the contrary, a significant (*p* < 0.0001 vs. CTRL) cytotoxic effect was observed for ciclopirox and actinomycin D, although to a different extent. Indeed, when tested at lower concentrations, ciclopirox was revealed to be more toxic, causing a higher reduction of IB3-1 cells survival (ranging from 47.7% at 1/4xMIC to 99% at 1/2xMIC) (Figure 10B) compared with actinomycin D (ranging from 38% at 1xMIC to 56.7% at 2xMIC) (Figure 10C).

## 3. Discussion

Repurposing or repositioning FDA-approved pharmacotherapies for off-label use has recently supplied alternative approaches to identifying new classes of antibiotics and scaffolds to combat infections caused by MDR pathogens [26]. Herein, we screened eight FDA-approved “non-antibiotic” drugs for their potential use in treating lung infections caused by *P. aeruginosa* in CF patients. With this aim, we tested six *P. aeruginosa* CF strains selected because of MDR and high-biofilm production. First, we evaluated the activity of each drug against *P. aeruginosa* planktonic cells. Ciclopirox, followed by 5-fluorouracil, and actinomycin D were the only ones showing antibacterial activity. 

A literature survey revealed that although these compounds had reported bioactivities, no study was focused on the antibacterial activity against *P. aeruginosa* in CF patients. Ciclopirox is an off-patent, broad-spectrum antifungal agent used, as an olamine salt, in various formulations to treat superficial fungal infections [27]. It has recently been found to have considerable potential to act against both Gram-positive and Gram-negative bacterial pathogens [20,28]. The synthetic fluorinated pyrimidine 5-fluorocytosine is used as an antimycotic drug with the brand name Ancobon. It has successfully been used to treat fungal infections in CF patients, including a case of pulmonary candidiasis, without causing side effects [29]. Actinomycin D, an antitumor antibiotic that inhibits transcription, is one of the oldest chemotherapy drugs used to treat various types of cancer, such as Wilms tumor, rhabdomyosarcoma, Ewing’s sarcoma, trophoblastic neoplasm, testicular cancer, and certain types of ovarian cancer [30].

Our results showed that ciclopirox has antibacterial activity against *P. aeruginosa* CF strains, with MIC values ranging from 128 to 512 µg/mL. Our findings are consistent with previous studies focused on *Acinetobacter baumannii*, *Escherichia coli*, and *Klebsiella pneumoniae* clinical isolates, although the effect was more potent as indicated by an MIC range of 5–15 µg/mL [20]. Despite the multiple potential uses of ciclopirox, very little is known about its antibacterial mechanism, thus warranting further studies. In this frame, Conley et al. observed that ciclopirox acts against *E. coli* interfering with galactose metabolism and blocking LPS synthesis [28]. *E. coli* and *K. pneumoniae* preferentially use glucose, metabolizing it through the Embden–Meyerhof–Parnas pathway. In contrast, *Pseudomonas* species use glucose as a secondary carbon source and catabolize it using the Entner–Doudoroff pathway [28]. This differential use of glucose as a carbon source might explain why we observed *P. aeruginosa* has higher ciclopirox MICs than *E. coli* and *K. pneumoniae*. Ribavirin, oxyclozanide, meloxicam, furosemide, and toremifene did not show any activity at the tested concentrations, although higher than those previously revealed as effective [13,14,16,17]. 

Tobramycin, along with colistin and aztreonam, is the inhaled antibiotic currently used for treating *P. aeruginosa* CF lung infections [31]. However, when antibiotic therapy is administered continuously and for long periods, it can select for resistant *P. aeruginosa* strains [32]. The use of bactericidal rather than bacteriostatic agents as first-line therapy is therefore recommended because the eradication of microorganisms serves to curtail, although not avoid, the development of bacterial resistance. The results we obtained from time–kill analyses indicated that actinomycin D and ciclopirox exhibit a strain-dependent bactericidal activity. Indeed, both drugs were bacteriostatic against the Pa7 strain but bactericidal against the tobramycin-resistant PaPh32 strain, even until causing a decrease in the viability under the limit of detection without regrowth over monitored time. Conversely, 5-fluorouracil and tobramycin were bacteriostatic and allowed for regrowth, regardless of the strain tested.

Current CF treatment regimens involve not only aggressive use of high-dose antibiotics but also combination therapy. However, the limited number of available antibiotics makes it difficult to know what combination would be most effective in any clinical situation. In this frame, several non-antibiotic compounds were reported to synergize with antibiotics, offering a new direction for fighting emergent drug-resistant pathogens such as *P. aeruginosa*. In this regard, Ejim et al. [33] found that benserazide (a DOPA decarboxylase inhibitor to treat Parkinson’s disease) and loperamide (an opioid receptor agonist used to treat diarrhea) restore minocycline susceptibility in MDR *P. aeruginosa* strains. In another study, the antipsychotic agents levomepromazine and chlorpromazine exhibited synergy with polymyxin B against *P. aeruginosa*, providing potential chemical scaffolds for further drug development [34]. In the present study, we tested the activity of tobramycin combined with ciclopirox, actinomycin D, and 5-fluorouracil against both tobramycin-resistant and -susceptible *P. aeruginosa* CF strains. Unfortunately, no synergistic effect was found. Each combination indeed resulted in an additive effect, regardless of tobramycin-resistant and -susceptible strains.

The treatment of pulmonary bacterial infections in CF patients is significantly affected by adaptative strategies that allow pathogens to survive despite repeated, broad-spectrum courses of antibiotics [35]. Among these strategies, biofilm formation provides bacterial communities with both physical protection and reservoirs of phenotypically distinct subpopulations that withstand antimicrobials and immune responses [35]. Though existing antibiotic therapies have improved CF patients’ lung function, survival, and quality of life, the biofilm lifestyle represents a barrier limiting benefits. In the present study, the antibiofilm potential of all eight drugs was evaluated, for the first time, against 24 h old preformed, mature *P. aeruginosa* biofilms. Scientific literature is inconsistent and fragmented in this regard. Previous studies reported the efficacy of actinomycin D in preventing biofilm formation by *S. epidermidis* and methicillin-resistant *S*. *aureus* strains [36,37]. Conversely, ciclopirox was previously shown to be not active on biofilm formation by MDR *A. baumannii*, *E. coli*, and *K. pneumoniae* clinical isolates [28]. Toremifene prevented biofilm formation and eradicated preformed biofilms by the oral bacteria *Porphyromonas gingivalis* and *Streptococcus mutans* [15]. No study was focused on 5-fluorouracil, ribavirin, furosemide, and oxyclozanide. Overall, our findings indicated ciclopirox as the most effective, in some cases even more active than tobramycin. Ciclopirox always caused a significant dispersion of biofilm biomass, consisting of EPS and cells, along with a reduction of biofilm viability nearly to eradication. Similarly, actinomycin D and 5-fluorouracil reduced biofilm viability, although to a lesser extent; furthermore, they stimulated EPS production as suggested by increased biofilm biomass after exposure. The same trend was observed for tobramycin when tested at sub-inhibitory concentrations. The EPS hyperproduction could prevent eradicating cells persisting within biofilms despite repeated rounds of antibiotic treatment due to reduced antibiotic penetration and inhibition of phagocytosis and complement activation [38]. It is worth noting the activity of ribavirin, the only one among the drugs without influence on planktonic cell growth that caused a significant dispersion of preformed biofilm by *P. aeruginosa*. The lack of published studies warrants further studies to elucidate the underlying mechanism(s) of action. No antibiofilm activity was observed for meloxicam, previously reported to significantly inhibit *P. aeruginosa* PAO1 biofilm formation in a dose-dependent manner [17].

Although repurposing existing drugs offers the advantage of known safety and pharmacokinetic profiles, the cytotoxicity of these compounds is still to be investigated for novel applications. Therefore, the potential cytotoxic effect of the biologically active drugs ciclopirox, actinomycin D, 5-fluorouracil, and ribavirin was investigated, in comparison with tobramycin, against human bronchial CF cells. Ribavirin and 5-fluorouracil did not show any toxicity against CF bronchial cells, similarly to tobramycin. Despite earlier studies showing that ciclopirox has an excellent safety profile toxicity in several animal models after several types of administration [39], we found it has potential for cytotoxicity at concentrations lower than 1% commonly used for topical administration. Actinomycin D, a potent inducer of apoptosis, showed a cytotoxic potential comparable to ciclopirox, in agreement with the previously observed hepatic, blood, gastrointestinal, and immune-system-related toxicity [40]. Developing a novel aerosolized formulation of ciclopirox or actinomycin D would be beneficial since it could significantly reduce the amount of a drug related to a clinically relevant outcome, as already shown for NSAIDs pulmonary delivery [41]. Moreover, nanoparticulate formulations using biocompatible and biodegradable polymers could improve the residence time of the drug in the lung by supplying a depot delivery to the lung following nebulization. Specifically, the incorporation of PEG has been shown to increase the diffusion of nanoparticles through human mucoid surfaces [42], which might be particularly relevant in CF patients.

A drug repurposing strategy has also been successfully used to find antivirulence compounds able to decrease the potential damage produced by the pathogens to the host [43]. Unlike conventional antimicrobials, they act without affecting bacterial growth, reducing the chances of developing resistance. In this context, actinomycin D was previously considered a potential antivirulence agent against *S. aureus* due to the hemolysis inhibition [37], while ciclopirox was found to inhibit pyocyanin, although it increased pyoverdine production in *P. aeruginosa* [20]. Therefore, for the first time, we tested the effects of the biologically active drugs on the expression levels of selected *P. aeruginosa* virulence genes using real-time RT-qPCR. The exposure to each drug at 1/4xMIC generally caused increased expression of most genes tested, although to different extents. Specifically, the most advantageous pattern was observed for ribavirin, which increased only *mexA*, *mexC*, and *toxA* expression, while the worst one was associated with ciclopirox, which provoked hyperexpression of all genes but *mexC*. It is worth noting that actinomycin D caused down expression of *aprA* and *algD* codifying for the main virulence traits of *P. aeruginosa*, the alkaline protease and GDP-mannose 6-dehydrogenase involved in the alginate polymerization needed for adhesion and subsequent biofilm formation, respectively. Similarly, we observed that 5-fluorouracil and tobramycin reduced *aprA* and *algD* expression, respectively. The anticancer drug 5-fluorouracil was recently proposed for repurposing as a quorum sensing inhibitor in *P. aeruginosa* [44]. However, the isolation of drug-insensitive spontaneous mutants indicates that resistance mechanisms can emerge even under in vitro conditions where the targeted virulence factor(s) is not required for growth [44]. Conversely, we found that exposure to 5-fluorouracil caused the down expression of the QS mediator *lasI*, probably due to the high variability in antivirulence activities previously observed among CF strains [44].

## 4. Materials and Methods

### 4.1. Drugs

Ribavirin, toremifene, oxyclozanide, meloxicam, 5-fluorouracil, actinomycin D, furosemide, and ciclopirox were tested comparatively with the aminoglycoside antibiotic tobramycin commonly prescribed for the inhalation therapy of *P. aeruginosa* infection in CF patients. All drugs tested were purchased from Merck KGaA (Darmstadt, Germany). According to the manufacturer’s recommendations, stock solutions were prepared in dimethyl sulfoxide (DMSO; Merck KGaA) or reagent-grade water, aliquoted, and stored at −80 °C until use.

### 4.2. Bacterial Strains and Standardized Inoculum Preparation

Nineteen clonally distinct *P. aeruginosa* strains isolated from sputum samples of CF patients were initially enrolled in the study. Each strain was identified using MALDI-TOF mass spectrometry and then stored at −80 °C until it was cultured twice on Mueller–Hinton agar (MHA; Oxoid, Milan, Italy) to restore the original phenotype. A standardized inoculum was prepared for each strain, depending on the use.

#### 4.2.1. Biofilm Formation

Several colonies that grew overnight onto Tryptone Soya Agar (TSA; Oxoid) were resuspended in Trypticase Soy broth (TSB; Oxoid) and incubated at 37 °C under agitation (130 rpm). After 16 h of incubation, the broth culture was adjusted with sterile TSB to an optical density measured at 550 nm (OD_550_) of 1.0—corresponding to 1–4 × 10^8^ CFU/mL—and finally diluted 1:100 (vol/vol) in TSB.

#### 4.2.2. Drug Susceptibility Assays of Planktonic Cells

Several colonies that grew overnight onto TSA (Oxoid) were resuspended in sterile NaCl 0.9% (Fresenius Kabi Italia, Verona, Italy), adjusted to a final concentration of 1–2 × 10^8^ CFU/mL, and finally diluted 1:1000 (vol/vol) in cation-adjusted Mueller–Hinton II broth (CAMHB; Becton, Dickinson & Co., Milan, Italy).

### 4.3. Biofilm Formation Assay

Two hundred microliters of the standardized inoculum were aseptically added to each well of a 96-well polystyrene tissue culture plate (Falcon BD; Becton, Dickinson & Co.). Negative controls were prepared similarly using TSB only. After 24 h of incubation at 37 °C under static conditions, biofilms were washed twice with PBS (pH 7.2) (Merck KGaA) to remove non-adherent cells and then fixed at 60 °C for 1 h. Biofilm biomass was stained for 5 min with 200 µL Hucker-modified crystal violet [45] and air-dried (37 °C, 30 min). Finally, crystal violet was extracted by exposure for 15 min to 200 μL of 33% glacial acetic acid (Merck KGaA). Biofilm biomass was measured as OD_492_ (Sunrise; Tecan, Milan, Italy). Based on the efficiency in biofilm formation, each strain was classified as follows [21]: non-biofilm-former (NBF) (OD ≤ ODc); weak biofilm-former (WBF) [ODc < OD ≤ (2xODc)]; moderate biofilm-former (MBF) [(2xODc) < OD ≤ (4xODc)]; and high biofilm-former (HBF) (OD > 4xODc). The cut-off value (ODc) for biofilm formation was defined as the mean OD of negative controls + 3x standard deviation.

### 4.4. Drug Susceptibility Assays of Planktonic Cells

The in vitro susceptibility of *P. aeruginosa* was evaluated, comparatively to tobramycin, using several assays.

#### 4.4.1. Disk Diffusion Assay

The susceptibility of *P. aeruginosa* isolates to several antibiotics (i.e., amikacin, ticarcillin, piperacillin/tazobactam, ceftazidime, gentamicin, netilmicin, levofloxacin, and tobramycin) was evaluated by the disk diffusion technique according to the CLSI guidelines [24] and using Multodisc Pseudomonas (Liofilchem Srl, Roseto degli Abruzzi, Italy). A strain was defined as multidrug-resistant (MDR) if non-susceptible to at least one agent in three or more antimicrobial categories among those tested (aminoglycosides, penicillins, cephalosporins, carbapenems, and fluoroquinolones) [22]. *E. coli* ATCC25922 and *P. aeruginosa* ATCC27853 were used as quality control strains.

#### 4.4.2. MIC and MBC Measurements

MIC and MBC values of each “non-antibiotic” drug were measured against 6 *P. aeruginosa* isolates, selected as representatives for MDR and HBF phenotypes. MIC was measured by the broth microdilution technique, according to the CLSI guidelines [24]. *E. coli* ATCC25922 and *P. aeruginosa* ATCC27853 were used as quality control strains. MBC was evaluated by plating onto MHA (Oxoid) 10 μL of broth culture from wells showing no visible growth at MIC determination. Following incubation at 37 °C for 24 h, the MBC value was defined as the minimum antibiotic concentration needed to eradicate 99.9% of the starting inoculum. Differences between MIC or MBC values were significant for discrepancies ≥2 log_2_ concentration steps.

#### 4.4.3. Time–Kill Assay

Kill kinetics of actinomycin, 5-fluorouracil, ciclopirox, and tobramycin against selected *P. aeruginosa* strains were evaluated by broth macrodilution. Briefly, the standardized inoculum (1–2 × 10^5^ CFU/mL) was exposed to several concentrations of each drug in CAMHB and incubated at 37 °C. At prefixed times (1, 2, 3, 4, 5, 6, 12, 16, 20, and 24 h), a cell viable cell count was performed, and the results were expressed by plotting Log (CFU/mL) over time, considering 10 CFU/mL as the LOD. Control samples were prepared similarly but were not exposed to drugs. The carry-over antibiotic effect was not observed. Bactericidal activity was defined as a ≥ 3 Log (CFU/mL) reduction.

#### 4.4.4. Checkerboard Microdilution Assay

The activity of tobramycin combined with 5-fluorouracil, ciclopirox, or actinomycin D was assessed against selected *P. aeruginosa* strains by the checkerboard microdilution method [25]. The fractional inhibitory concentration index (FICi) was calculated as FIC_A_ + FIC_B_, where FIC of drug A (FIC_A_) = MIC of drug A in combination/MIC of drug A alone and FIC of drug B (FIC_B_) = MIC of drug B in combination/MIC of drug B alone. FICi was and interpreted as follows: synergy, FICi ≤ 0.5; additivity, 0.5 < FICi ≤ 4; indifference, FICi = 2; antagonism, FICi > 4 [25].

### 4.5. In Vitro Activity against Preformed Biofilms

Biofilms were grown for 24 h in a 96-well microtiter plate as previously described in “Biofilm formation assay”. Next, they were exposed to each drug tested at the desired concentrations prepared in CAMHB. Following 24 h of exposure at 37 °C under static conditions, the effect against mature biofilms was evaluated in terms of biofilm biomass dispersion (crystal violet assay, as described in “Biofilm formation assay”) and residual viability. In the latter case, non-adherent bacteria were removed after drug exposure by washing once with sterile PBS, then biofilm samples were scraped following a 5 min exposure to 100 μL trypsin-ethylenediaminetetraacetic acid 0.25% (Merck KGaA), and finally the suspension underwent to viable cell count on MHA. The percentage of inhibition of biofilm formation or dispersal of preformed biofilms following drug exposure was calculated as follows: (i) (1 – OD_492_ of test/OD_492_ of untreated control) ×100, in the case of crystal violet assay; (ii) [(CFU/well of the test)/(CFU/well of untreated control)] ×100, in the case of plate count assay.

### 4.6. Gene Expression Assay

The effect of drug exposure on the transcription levels of *algD*, *toxA*, *lasI*, *aprA*, *mexA*, *mexB*, and *mexC* virulence genes by *P. aeruginosa* PaPh32 was assessed by real-time reverse transcription quantitative PCR (RT-qPCR). Planktonic cells were exposed to each drug at 1/4xMIC for 20 h at 37 °C, washed with PBS, and then harvested in Qiazol (Qiagen; Milan, Italy). RNA was extracted by the phenol–chloroform technique, treated with DNase I (Merck KGaA), and checked for purity and quantity by NanoDrop-2000 spectrophotometer (Thermo Fisher Scientific Italia Inc., Monza, Italy). Strand cDNA was synthesized from 2 µg of RNA using a high-capacity cDNA reverse transcription kit (Thermo Fisher Scientific Italia), and gene expression was then evaluated using 10 ng of cDNA by RT-qPCR assay on QuantStudioTM 7 Pro Real-Time PCR System (Applied Biosystems) using the PowerTrack SYBR Green Master Mix (Thermo Fisher Scientific Italia Inc.). Primers were designed using as a reference the genome of *P. aeruginosa* strain NDTH9845 (GenBank accession number: CP073080.1) (Table 5).

Specificity was assessed in silico with BLAST and by PCR endpoint under the same real-time RT-qPCR conditions. The ΔΔCt method was applied to evaluate the relative gene expression in exposed vs. unexposed cells after normalizing on the *proC* housekeeping gene expression. The modulation of expression levels was shown as fold change.

### 4.7. Cytotoxicity Evaluation

The cytotoxic effect of each drug was assessed towards IB3-1 bronchial epithelial cells (ATCC#CRL-2777) isolated from a pediatric CF patient who harbored the ΔF508/W1282X mutations within the CFTR gene. Cells were grown as a monolayer at 37 °C in LHC-8 medium (Thermo Fisher Scientific Italia) supplemented with 5% fetal bovine serum (Gibco, Milan, Italy) in a 5% CO_2_ atmosphere. After exposing the monolayer to each drug at the desired concentration for 24 h, the cell viability was measured by an MTS tetrazolium-based colorimetric assay (CellTiter 96^®^ AQueous One Solution Cell Proliferation Assay; Promega, Milan, Italy). Briefly, 20 μL of a mixture of MTS [3-(4,5-dimethylthiazol-2-yl)-5-(3-carboxymethoxyphenyl)-2-(4-sulfophenyl)-2H-tetrazolium] and the electron coupling reagent PES (phenazine ethosulfate) were added to each well containing exposed cells. Untreated IB3-1 cells were used as control. After 4 h of incubation at 37 °C, the OD_492_ was measured using the ELISA plate reader Sunrise (Tecan, Männedorf, Switzerland).

### 4.8. Statistical Analysis

Each experiment was carried out at least in triplicate and repeated on two different occasions (*n* ≥ 6). Statistical analysis was performed using GraphPad software (ver. 8.0; GraphPad Inc., San Diego, CA, USA). Data distribution was assessed using the D’Agostino and Pearson normality test, and then the differences in the biofilm biomass (OD_492_) were evaluated using: (i) ANOVA + Tukey’s multiple comparisons post-test for datasets normally distributed; (ii) ordinary one-way ANOVA + Holm–Sidak’s multiple comparisons post-test in case datasets did not pass the normality test. Differences between percentages were assessed using Fisher’s exact test. The significance level was set at *p* < 0.05.

## 5. Conclusions

Overall, our findings indicated ciclopirox and ribavirin as attractive candidates for repurposing as anti-*P. aeruginosa* agents in CF patients. Ciclopirox exhibited relevant antibacterial and antibiofilm activities, although further studies are needed to decrease its cytotoxic potential. At safe concentrations, ribavirin, a guanosine analog with broad-spectrum virustatic activity, showed a specific antibiofilm effect since it was not related to antibacterial activity. In addition, it has already received FDA approval as an aerosol formulation, although for respiratory syncytial virus-infected infants. Ciclopirox and ribavirin have also been shown to have anti-inflammatory [47] and immunomodulatory [48] properties, respectively, which is highly relevant in CF patients where an exuberant, acute inflammatory response leads to pulmonary tissue damage and failure in clearing the infection [1,2].

Ciclopirox and ribavirin might therefore represent a good starting point for traditional medicinal chemistry providing potential chemical scaffolds for further drug development. Pre-clinical studies are warranted to evaluate the protective effect in animal models and pharmacodynamics/pharmacokinetics in humans.

## Figures and Tables

**Table 1:** FDA-approved “non-antibiotic” drugs tested. We selected some in-use pharmaceuticals found to have some direct antimicrobial effects in previously published studies.

Drug	Therapeutic Class	Clinical Use	Cat. No. ^a^	Solvent ^b^	[Stock]	Antibacterial Spectrum	Antibacterial Activity ^c^	Reference
Ribavirin	Nucleoside analogue	Chronic hepatitis C and other flavivirus infections	R9644	H_2_O	10 mg/mL	*P. aeruginosa*	3000	[13,14]
Toremifene	Nonsteroidal antiestrogen	Several types of cancer	T7204	H_2_O, DMSO	3.3 mg/mL	*S. aureus* *P. gingivalis* *S. mutans*	12.5–25	[14,15]
Oxyclozanide	Salicylanilide anthelmintic	Fascioliasis in ruminants	34078	DMSO	50 mg/mL	*P. aeruginosa*	256	[16]
Meloxicam	Nonsteroidal anti-inflammatory	Pain and inflammation in rheumatic diseases and osteoarthritis	M3935	H_2_O	10 mg/mL	*P. aeruginosa*	31	[17]
5-Fluorouracil	Pyrimidine analog	Several types of cancer	F6627	DMSO	50 mg/mL	*S. aureus*	4	[14]
Actinomycin D	Actinomycins	Several types of cancer	A1410	DMSO	4 mg/mL	*P. aeruginosa* *S. aureus*	0.06–32	[13,14,18,19]
Furosemide	Loop diuretic	Peripheral, pulmonary, and cerebral edema; hypertension	PHR1057	DMSO	50 mg/mL	*P. aeruginosa*	50% biofilm reduction at 10 µg/mL	[13,14]
Ciclopirox	Antifungal	Surface fungal infections	SML2011	DMSO	50 mg/mL	*P. aeruginosa*	30	[20]

^a^ All drugs were from Merck KGaA (Darmstadt, Germany). ^b^ H_2_O: reagent-grade water; DMSO: dimethyl sulfoxide. ^c^ Values are MIC (µg/mL), except for furosemide.

**Table 2:** Antibiotic susceptibility of 19 *P. aeruginosa* strains. The activity of amikacin (AK), ticarcillin (TC), piperacillin/tazobactam (TZP), ceftazidime (CAZ), colistin (CN), netilmicin (NET), levofloxacin (LEV), and tobramycin (TOB) was measured by the disk diffusion agar method. The inhibition zone diameter values were shown and interpreted according to CLSI guidelines [24]: resistance is highlighted in red and susceptibility is highlighted in green, while intermediate susceptibility is not highlighted. A strain was defined as multidrug-resistant (MDR) if non-susceptible to at least one agent in three or more antimicrobial categories among those tested [22].

Strains	AK	TC	TZP	CAZ	CN	NET	LEV	TOB	MDR
Pa2	0	0	14	0	0	0	13	0	Yes
Pa3	0	0	19	0	0	0	11	0	Yes
Pa4	24	0	0	0	0	0	25	0	Yes
Pa5	11	0	0	0	10	0	23	13	Yes
Pa6	15	0	0	0	14	10	22	17	Yes
Pa7	13	0	0	0	12	8	25	15	Yes
Pa9	0	0	0	0	0	0	0	0	Yes
Pa10	0	0	0	0	0	0	11	0	Yes
Pa14	30	0	16	8	20	19	29	21	Not
Pa16	23	32	27	14	20	16	19	24	Not
Pa21	18	8	13	0	15	11	23	24	Yes
Pa41	21	8	8	18	15	7	21	24	Yes
PaPh13	25	10	18	10	18	16	27	24	Yes
PaPh14	18	0	11	0	15	14	25	24	Yes
PaPh26	15	20	20	0	16	16	0	24	Yes
PaPh32	20	0	15	9	0	13	0	7	Yes
DIN1	26	0	13	0	21	19	18	23	Yes
AC12a	18	13	19	12	16	13	25	24	Yes
PaM	24	0	0	0	24	22	9	24	Yes

**Table 3:** Susceptibility of *P. aeruginosa* CF strains to FDA-approved drugs. MIC and MBC values were measured, comparatively to tobramycin, by the broth microdilution method and expressed as µg/mL. Differences in the range of tested concentrations are due to limitations in drugs’ solubility. ^a^ Tobramycin-resistant strains are underlined.

Drugs	*P. aeruginosa* Strains ^a^											
	Pa5		Pa6		Pa7		Pa41		PaPh32		DIN 1	
	MIC	MBC	MIC	MBC	MIC	MBC	MIC	MBC	MIC	MBC	MIC	MBC
**Ribavirin**	>1024	>1024	>1024	>1024	>1024	>1024	>1024	>1024	>1024	>1024	>1024	>1024
**Oxyclozanide**	>1024	>1024	>1024	>1024	>1024	>1024	>1024	>1024	>1024	>1024	>1024	>1024
**Meloxicam**	>1024	>1024	>1024	>1024	>1024	>1024	>1024	>1024	>1024	>1024	>1024	>1024
**5-Fluorouracil**	128	>1024	256	>1024	128	>1024	>1024	>1024	512	1024	>1024	>1024
**Furosemide**	>1024	>1024	>1024	>1024	>1024	>1024	>1024	>1024	>1024	>1024	>1024	>1024
**Ciclopirox**	128	>1024	256	>1024	128	>1024	512	>1024	256	>1024	512	>1024
**Toremifene**	>330	>330	>330	>330	>330	>330	>330	>330	>330	>330	>330	>330
**Actinomycin D**	266	>266	266	>266	266	>266	>266	>266	133	133	266	266
**Tobramycin**	8	16	2	4	2	4	0.5	1	64	>64	0.5	0.5

**Table 4:** Activity of tobramycin in combination with other “non-antibiotic” drugs. The fractional inhibitory concentration index (FICi) was calculated as follows, using the checkerboard assay: FIC_A_ + FIC_B_, where FIC_A_ = MIC of drug A in combination/MIC of drug A alone, and FIC_B_ = MIC of drug B in combination/MIC of drug B alone. The best FICi value and the range of FICi values were reported for each drug combination. All FICi values obtained indicated an additive effect (0.5 < FICi ≤ 4) [25].

Drug Combinations	Best FICi Value (Range) for *P. aeruginosa* Strain:	
	Pa7	PaPh32
Tobramycin + actinomycin D	0.63 (0.63–1.06)	0.75 (0.75–1.25)
Tobramycin + ciclopirox	0.56 (0.56–1.25)	0.63 (0.63–1.25)
Tobramycin + 5-fluorouracil	1 (1–2.25)	1.13 (1.13–4.25)

**Table 5:** Primer sequences used in real-time reverse transcription quantitative PCR analyses. The expression of selected virulence genes by *P. aeruginosa* PaPh32 was evaluated after 20 h of exposure to FDA-approved drugs by real-time RT-qPCR using oligonucleotides designed on the sequence of *P. aeruginosa* strain NDTH9845 (GenBank accession number: CP073080.1). The gene *proC* was used as housekeeping [46].

Target Gene	Primer Sequences	RT-qPCR Product (bp)	Gene Function
*algD*	F: 5′-CGACCTGGACCTGGGCTAC-3′	144	Alginate
	R: 5′-TCCTCGATCAGCGGGATC-3′		
*toxA*	F: 5′-TGGAGCGCAACTATCCCAC-3′	148	Exotoxin A
	R: 5′-TAGCCGACGAACACATAGCC-3′		
*lasI*	F: 5′-GAGCTTCTGCACGGCAAGG-3′	68	Quorum sensing
	R: 5′-TTGATGGCGAAACGGCTGAG-3′		
*aprA*	F: 5′-TACCTGATCAACAGCAGCTACAG-3′	195	Alkaline protease
	R: 5′-GTAGCTCATCACCGAATAGGCG-3′		
*mexA*	F: 5′-AGCAAGCAGCAGTACGCC-3′	86	Efflux pump
	R: 5′-GTGTAGCGCAGGTTGATCC-3′		
*mexB*	F: 5′-GCCTCGATCCATGAGGTAGTG-3	74	Efflux pump
	R: 5′-AGGAACAGGTACATCACCAGG-3′		
*mexC*	F: 5′-ACGTCGGCGAACTGCAAC-3′	101	Efflux pump
	R: 5′-CTGAAGAAAGGCACCTTGGC-3′		
*proC*	F: 5′-AGGCCGGGCAGTTGCTGTC-3′	178	Proline biosynthesis
	R: 5′-GTCAGGCGCGAGGCTGTC-3′		

## Data Availability

Not applicable.
